# How do patients with inflammatory bowel disease want their biological therapy administered?

**DOI:** 10.1186/1471-230X-10-1

**Published:** 2010-01-10

**Authors:** Patrick B Allen, Hannah Lindsay, Tony CK Tham

**Affiliations:** 1 Division of Gastroenterology, Ulster Hospital, Dundonald, Belfast, N Ireland, BT16 1RH, UK; 2 Department of Quality and Effectiveness, Ulster Hospital, Dundonald, Belfast, N Ireland, BT16 1RH, UK

## Abstract

**Background:**

Infliximab is usually administered by two monthly intravenous (iv) infusions, therefore requiring visits to hospital. Adalimumab is administered by self subcutaneous (sc) injections every other week. Both of these anti-TNF drugs appear to be equally efficacious in the treatment of Crohn's Disease and therefore the decision regarding which drug to choose will depend to some extent on patient choice, which may be based on the mode of administration.

The aims of this study were to compare preferences in Inflammatory Bowel Disease (IBD) patients for two currently available anti-TNF agents and the reasons for their choices.

**Methods:**

An anonymous questionnaire was distributed to IBD patients who had attended the Gastroenterology service (Ulster Hospital, Dundonald, Belfast, N. Ireland. UK) between January 2007 and December 2007. The patients were asked in a hypothetical situation if the following administering methods of anti-TNF drugs (intravenous or subcutaneous) were available, which drug route of administration would they choose.

**Results:**

One hundred and twenty-five patients fulfilled the inclusion criteria and were issued questionnaires, of these 78 questionnaires were returned (62 percent response). The mean age of respondent was 44 years. Of the total number of respondents, 33 patients (42 percent) preferred infliximab and 19 patients (24 percent) preferred adalimumab (p = 0.07). Twenty-six patients (33 percent) did not indicate a preference for either biological therapy and were not included in the final analysis. The commonest reason cited for those who chose infliximab (iv) was: *"I do not like the idea of self-injecting," *(67 percent). For those patients who preferred adalimumab (sc) the commonest reason cited was: *"I prefer the convenience of injecting at home," *(79 percent). Of those patients who had previously been treated with an anti-TNF therapy (n = 10, all infliximab) six patients stated that they would prefer infliximab if given the choice in the future (p = 0.75).

**Conclusions:**

There was a trend towards patient preference for infliximab (iv) treatment as opposed to adalimumab (sc) in patients with IBD. This difference may be due to the frequency of administration, mode of administration or differing 'times in the market-place', as infliximab had been approved for a longer period of time in Crohn's disease. Further studies are required in IBD patients to investigate whether patient choice will affect compliance, patient satisfaction and efficacy of treatment with anti-TNF therapies.

## Background

Inflammatory bowel diseases (IBD) are chronic relapsing and remitting disorders with varying degrees of severity. The early use of biological therapies, in particular anti-TNF therapies (e.g. infliximab and adalimumab) can induce and significantly increase remission rates without the need for corticosteroids and surgery in Crohn's disease [1]. In moderate-to-severe Ulcerative Colitis, infliximab has been shown to significantly reduce the rate of colectomy [2]. Furthermore infliximab may alter the natural history of Crohn's disease post-operatively by reducing endoscopic and histological recurrence [3]. Adalimumab is a relatively new anti-TNF therapy and is also licensed for the treatment of Crohn's disease. It appears to be effective in inducing remission in Crohn's disease [4] and has been shown to reduce hospitalisations and surgery at one year [5], with resulting sustained improvements in health-related quality of life [6]. Therefore with the potential exciting benefits of anti-TNF therapy there may be a further increase in the use of these drugs in the future management of IBD.

These drugs have contrasting modes of administration and dosing schedules. Infliximab (5 mg/kg maintenance; Remicade^®^, Schering-Plough) is administered by intravenous (iv) infusion usually once every six to eight weeks in a day-treatment facility by a trained healthcare professional, over a period of two hours. Patients are required to stay for up to one to two hours after an infusion for clinical observation. Adalimumab (40 mg maintenance, Humira^®^, Abbott Laboratories) was approved for use in Crohn's Disease in 2007 and in contrast to infliximab, is administered by subcutaneous (sc) injection usually once every two weeks.

These two anti-TNF drugs while appearing to have similar efficacies, have contrasting modes of administration and may offer potential opportunities to patients. The advantages of self-administration may be that patients do not need to attend clinics at specific times, allowing flexibility of administration. But this will require the patient or a close family member to be responsible for administration. In contrast infliximab requires regular attendance at a day case treatment facility, but the patient and/or family member have minimal responsibilities for the administration of the drug. This attendance provides an opportunity for the patient and family to discuss concerns about their disease or treatment with either other patients or a healthcare professional.

Shared decision making, in which patients and health professionals join in both the process and responsibility for the decisions made, is attracting considerable interest as a strategy by which patients' preferences can be employed into management decisions [7]. Where several treatment options exist which may have different effects on the patients quality of life, there is a strong argument to offer patients the choice of agent as their active involvement in decision making may increase the effectiveness of the treatment [8]. This is supported by *The UK Department of Health Guidance *in 2001[9], which recommends that patients should be fully involved in the management decisions of *'non life-threatening diseases'*.

It is thought that improved awareness of the factors that influence patient preferences will facilitate the patient-doctor communication, discussion and ultimately improve patient management [10]. Hibbard has stated that patients' preferences should be considered as importantly as a *'vital sign'*, and therefore should be regularly monitored and attended to by clinicians [11]. A previous study which examined the 'weight' of patient preference on final prescribing, showed that 50 percent of patients wished to leave final medical decisions to the clinician but 96 percent ultimately wanted to be offered choices and asked their opinion [12].

Patient preference studies comparing drugs of the same class but having differing dosing frequencies have been performed in various disease states including: diabetes (e.g. comparing differing modes of insulin delivery) [13]; gastro-esophageal reflux disease [14]; osteoporosis [15-19] and irritable bowel syndrome [20]. However patient preference studies for anti-TNF drugs are limited and have only previously been reported in the setting of rheumatoid arthritis [21]. No previous studies have been performed in IBD patients to assess preference for drug treatments, and in particular preference for anti-TNF therapies.

The aims of this study are to compare the preferences in IBD patients for two currently available anti-TNF agents in terms of their mode of administration, and the reasons for their choices.

## Methods

The inclusion criteria for this study were IBD patients who had attended the Ulster Hospital (Dundonald, Belfast, NI. UK) Gastroenterology Service (either as an in- or out-patient consultation) during the period January 2007 to December 2007. Patients were identified from the Ulster Hospital Inflammatory Bowel Disease Database, (Rotherham IBD database, Ferring Pharmaceuticals).

A questionnaire was devised and contained the following questions: age of patient; their principal diagnosis (Crohn's, ulcerative colitis, or indeterminate colitis); whether they have ever required surgery for complications and if so how many procedures they had undergone. The questionnaire also included: the number of visits to the Gastroenterology Service in the previous 12 months; and what impact IBD has had on (a) lifestyle and (b) employment. The patients self-reported their list of medications both currently and previously prescribed [Appendix 1].

The patients were then asked in a hypothetical scenario as to which route of administration of anti-TNF therapy that they would prefer if given the choice in the future. The possible outcomes were as follows: (a) infliximab [iv] in hospital every 8 weeks approximately, (b) adalimumab [sc] every 2 weeks at home, or (c) no preference for biological therapy. The patients were asked for the reasons for their choice of particular administration route. In addition, patients who had previously received an anti-TNF therapy were asked whether they would choose to have the same or alternative route of administration in the future if indicated.

### Statistical Analyses

The responses were analysed using the Binomial Probability test (Statistical Package for the Social Sciences [SPSS], Version 14) to analyse potential differences in preference for anti-TNF therapies, a p value of < 0.05 was considered statistically significant.

This questionnaire study was approved for use by the 'Quality and Effectiveness Department' at the Ulster Hospital, Dundonald, Belfast.

## Results

One hundred and twenty-five patients fulfilled the inclusion criteria and were issued questionnaires, of these 78 questionnaires were returned, (62 percent response). Please refer to table 1 for: average age of respondent; diagnoses; the length of course of the disease; the number of surgeries; and the number of visits to outpatient department in the previous twelve months.

**Table 1:** The number of patients included in study, time from diagnosis (years), requirements for surgery and those who had attended the Gastroenterology Outpatients in 2007.

		Number of respondents (%)
		
		n = 78
**Mean age of respondent**		**44 years**

**Diagnoses**	Ulcerative Colitis	40 (51%)
	
	Crohn's Disease	28 (36%)
	
	Indeterminate colitis	10 (13%)

**Time from diagnosis (years)**	<1 year	1 (2.6%)
	
	1-3 years	19 (24.4%)
	
	4-7 years	24 (30%)
	
	8-10 years	10 (12.8%)
	
	>10 years	24 (30%)

**Requirement(s) for surgery**	Total number of patients who had previous surgery	18 (23%)
	
	1 operation	9 (47%)
	
	2 operations	1 (6%)
	
	3 operations	2 (12%)
	
	4 or more operations	6 (35%)

**No. of patients attending the Gastroenterology Outpatient Department in previous 12 months**	One visit	24 (31%)
	
	two visits	18 (23%)
	
	three or more visits	22 (28%)

The medications currently and previously prescribed were collated. In total 59 patients (75 percent) had previously or were currently prescribed 5-aminosalicylates (5-ASA). In total, ten patients (13 percent) had previously been prescribed biological therapies (n = 10 all infliximab).

In response to the question, *"How much has your lifestyle been affected by inflammatory bowel disease?" *The responses were as follows: *"Not at all," *(46 percent); *"A moderate influence" *(23 percent) and; *"A great deal of influence" *(31 percent). In response to the question, *"How much influence has IBD had on your employment?" *The responses were: *"Not at all," *(33 percent); *"A moderate influence" *(29 percent); and, *"A great deal of influence" *(38 percent).

The patients were asked in a hypothetical situation as to which mode of administration of anti-TNF agent they would prefer if given the choice. Of the total number of respondents, 33 patients (42 percent) preferred infliximab and 19 patients (24 percent) preferred adalimumab (p = 0.07). Twenty-six patients (33 percent) did not indicate a preference for either biological therapy; these patients were not included in the analysis.

The commonest reasons cited for those patients who chose infliximab were: *"I do not like the idea of self-injecting," *(n = 22, 67 percent); *"I prefer to take the drug less often," *(n = 14, 42 percent); and, *"I prefer the convenience of this choice*," (n = 12, 36 percent). The commonest reasons cited for those patients who chose adalimumab were: *"I prefer the convenience of injecting at home," *(n = 15, 79 percent); *"No requirement to visit hospitals regularly between clinic visits," *(n = 12, 63 percent); and *"I prefer the less complicated technique of drug administration*," (n = 10, 53 percent).

Of the patients who had previously or currently receiving infliximab (i.e. n = 10), six stated that would prefer infliximab if given the choice in the future. This difference was not statistically significant (p = 0.75).

## Discussion and Conclusions

In this study nearly twice the number of patients surveyed would prefer to have an intravenous infusion (two-monthly) as opposed to self-administering a subcutaneous injection at home (fortnightly), however this difference did not reach the pre-specified level of 0.05. This may be due to the small sample size and the study may be underpowered as a consequence. A third of patients did not indicate any preference for either anti-TNF therapy and therefore we could assume that they would be satisfied with either mode of administration. In this study there were no significant differences in preference for either anti-TNF therapy in those that had previously received infliximab, although these were a small number of patients.

If we assume that these two drugs have comparable efficacy in IBD patients then there are some important differences between them. These are as follows: (1) infliximab requires intravenous infusion and therefore can only be given in a hospital setting as opposed to adalimumab; (2) infliximab requires less frequent dosing i.e. usually every eight weeks as opposed to every two weeks for adalimumab; and (3) infliximab has been licensed for a considerably longer period (FDA approved in 1998) for IBD patients than adalimumab (FDA approved in 2007).

Preference for less frequent drug dosing has been observed in other comparative studies in patients with osteoporosis [15-18]. In one study in patients with osteoporosis the most important reasons for drug preference derived from 'importance ratings' were the: drug effectiveness (79 percent of patients ranked it as their number one priority); time on market (14 percent); dosing procedure (4 percent) and lastly, dosing frequency (3 percent) [19].

This is interesting as it appears that when preference for two drugs with similar efficacy are compared in patients with osteoporosis, the time on market appears to be an important influencing factor in their rationale. This may have accounted for the trend in preference for infliximab in this study as it has been licensed for a greater time on the market than adalimumab.

In contrast to patients with osteoporosis [19], it appears that frequency of dosing may be an important determinant in treatment preference decisions in patients with IBD. As previously stated nearly twice the number of patients chose infliximab over adalimumab, and of these 42 percent of patients cited *'less frequent injections' *as the reason for their choice.

In this study the dosing procedure appeared to be important in the treatment decisions in IBD patients as a large proportion that chose infliximab (67 percent), stated that they did not like 'self-injecting administration'. This is in contrast to osteoporosis patients where the dosing procedure appears to be less relevant [19].

Patient preference studies for anti-TNF therapies have only been reported in patients with Rheumatoid arthritis (RA). The findings from this study are in contrast to these previous studies which addressed preferences for anti-TNF therapy in RA patients. In one study significantly more patients preferred adalimumab to infliximab [21]. The reasons patients cited for preferring adalimumab were that it was convenient to administer and would allow them to regain control over their lives. In another study, one hundred consecutive RA patients were surveyed as to which anti-TNF therapy they would prefer if given the choice. The results were similar in that most patients preferred the subcutaneous rather than intravenous route of administration and the majority of patients indicated that they would prefer to receive treatment at home rather than in hospital [22].

It is unclear why patients with RA and IBD differ in their preferences for biological therapy. Some possible explanations may include the following: (1) RA patients may be more immobile and therefore have greater difficulty in attending hospital and therefore would be happier to self-administer at home; (2) Adalimumab has been licensed for a longer period of time in RA, and these patients may have more experience or knowledge of its use than IBD patients; and (3) many Rheumatology Units have dedicated Rheumatology nurses available to administer these biological drugs, and to instruct and counsel patients. This scenario of provision of specialist nurses is not the reality in many IBD units in the UK as evidenced by the *UK Inflammatory Bowel Disease Audit in 2006 *which highlighted the inadequate provision of dedicated IBD nurses [23]. Rheumatology patients may feel that they have better contact with the medical and/or nursing team if required and therefore may be better educated and prepared to self-administer anti-TNF drugs at home.

Another possible explanation in this study for the trend in preference towards infliximab is that a large proportion of patients (i.e. 73 percent) have had their disease for greater than four years, and therefore may have more knowledge of its use in IBD.

Interestingly a large proportion of IBD patients in this study, believed that their disease had a 'moderate' to 'a great deal of influence' on their lifestyle and employment. The quality of life issues in inflammatory bowel disease and disease-related quality of life scoring indexes are well documented [24,25] and there is evidence of the beneficial effects of anti-TNF therapy on quality of life [6]. Disability in inflammatory bowel disease is not clearly defined except for work-related absences [26]. The majority of these patients have stated that they are unable to perform normally at work and their disease affects their lifestyle, implying that they may be 'functionally-impaired'. The ability of a patient to be treated early in their disease course, thereby possibly preventing future complications may affect their quality of life and reduce their work-related and other disabilities. Validated measures of disability require further studies in patients with inflammatory bowel disease and are currently being addressed by the World Health Organisation.

Patient-involved decision making with regard to the route of administration of anti-TNF therapy may improve compliance and ultimately success of therapy [8]. It has previously been shown that patients are more likely to participate in decision making regarding their medications when they understand the various alternatives [27]. In relation to the potential success of therapy an advantage of infliximab is that it may be preferable in those with poor compliance allowing health professionals to better observe these patients. In contrast adalimumab may have the potential advantages of allowing better patient autonomy and may prove to be a lesser economic burden on the provision of health care services [28].

The limitations of this study were the small number of patients included and only a small number had previously received anti-TNF therapies. This may have accounted for the non-significant differences obtained.

Further studies are needed in IBD patients to determine whether patient choices affect compliance, satisfaction and efficacy of treatment with anti-TNF therapies. As the efficacy and safety profile of infliximab and adalimumab appear to be similar, the decision regarding which anti-TNF therapy to use may be determined by joint physician-patient discussion and ultimately patient choice.

## Competing interests

TT is on the advisory boards for Schering-Plough and Abbott laboratories and has accepted hospitality from these two companies. PA has accepted hospitality from Schering-Plough and Abbott Laboratories. HL has no competing interests.

## Authors' contributions

All authors read and approved the final manuscript. PA carried out design of study and performed the statistical analysis, and drafting of the manuscript. HL helped with the design of the study, and distributed and collated the questionnaires. TT thought of the original idea for this study, assisted with the design of the study, drafting and revision of the manuscript.

## Pre-publication history

The pre-publication history for this paper can be accessed here:

http://www.biomedcentral.com/1471-230X/10/1/prepub
